# Pre-treatment evaluation of 5-fluorouracil degradation rate: association of poor and ultra-rapid metabolism with severe toxicity in a colorectal cancer patients cohort

**DOI:** 10.18632/oncotarget.7991

**Published:** 2016-03-08

**Authors:** Federica Mazzuca, Marina Borro, Andrea Botticelli, Eva Mazzotti, Luca Marchetti, Giovanna Gentile, Marco La Torre, Luana Lionetto, Maurizio Simmaco, Paolo Marchetti

**Affiliations:** ^1^ Oncology Unit, Sant'Andrea Hospital, Rome, Italy; ^2^ Department of Clinical and Molecular Medicine, Sapienza University of Rome, Rome, Italy; ^3^ Department of Neurosciences, Mental Health and Sensory Organs (NESMOS), Sapienza University of Rome, Rome, Italy; ^4^ Department of Clinical Oncology, Policlinico Umberto I, Rome, Italy; ^5^ Istituto Dermopatico dell'Immacolata-IRCCS, Rome, Italy

**Keywords:** 5-fluorouracil degradation rate, fluorouracil toxicity prediction, DPYD, colorectal cancer, polymorphisms

## Abstract

Despite the wide use of 5-fluorouracil-based chemotherapy, development of severe toxicity that follow the treatment is not a rare event. The efforts to establish pretreatment tools for toxicity prediction, led to the development of various pharmacogenetic and biochemical assays, mainly targeted to assess the activity level of dihydropyrimidine dehydrogenase (DPD), the main metabolizing enzyme for 5-fluorouracil. Using peripheral blood mononuclear cells, we developed a biochemical assay, that is not limited to the evaluation of DPD activity, but determines the net result of all the enzymatic transformation of 5FU, in terms of the amount of drug consumed by the cells in a time unit. This parameter, named 5-fluorauracil degradation rate, presents a normal distribution inside the population and highlight the presence of an ultra-rapid metabolizers class of subjects, besides the expected poor metabolizers class. Here we will show that, in a colorectal cancer patient cohort, both poor and ultra-rapid metabolizers have significantly increased the risk of developing severe toxicity (grade3–4). Patient stratification depending on the individual 5-fluorouracil degradation rate allows to identify a 10% of the overall population at high risk of developing severe toxicity, compared to the 1.3% (as assessed in the Italian population) identified by the most commonly employed pharmacogenetic test, including the DPD polymorphism IVS14+1G>A.

## INTRODUCTION

Fluorouracil, in combination with oxaliplatin, irinotecan, and biological agents, is the most common first line chemotherapy to treat colorectal cancer (CRC), both in the adjuvant and palliative setting, [1–3]. Despite efficacy, severe toxicity represents a major cause of reduced dosage, delayed drug administration and therapy discontinuation. Grade 3–4 toxicity is reported in about 30% of patients, with a 0.5% of toxic deaths [4, 5]. This figure means that every year the 5-FU toxicity determines approximately 1,300 deaths in the USA [6] and 200 in France or Italy [7]. Moreover, a higher number of patients suffer from unduly toxic effects, with avoidable suffering and reducible costs for the Health Systems.

Pre-emptive identification of patients who develop 5-FU severe toxicity is still an open issue in cancer management, hence the available methods identify only a small fraction of such patients.

**Figure 1:**
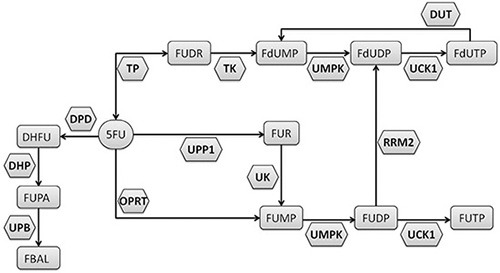
Metabolism of the 5-fluorouracil (5-FU)

The dihydropyrimidine dehydrogenase enzyme (DPD, encoded by the *DPYD* gene) transforms about 80% of the administrated 5-FU in the inactive metabolite 5, 6-dihydro-5-fluorouracil. The remaining 20% of the drug is catabolized by activating enzymes (Figure 1), with the production of metabolites accounting for inhibition of thymidylate synthase (*TYMS*) and for RNA/DNA damage [8]. An impaired activity of DPD leads to an increased production of cytotoxic metabolites and has been clearly associated with 5-FU induced severe toxicities [9–11].

The most used pharmacogenetic assay to predict DPD activity evaluates the presence of the splice site IVS14+1G>A polymorphism in the *DPYD* gene, which leads to production of a truncated, inactive protein and is associated with severe toxicity in about one half of carriers [12]. However, the IVS14+1G>A polymorphism has low frequency and it is not present in the majority of the patients with high 5-FU toxicity. Recently, we described a that *DPYD* haplotype is associated with a decreased value of 5FUDR and it could be related to toxicity development [13]. The functional effect of additional *DPYD* polymorphisms has been evaluated, but for the moment their prediction power results inadequate [14].

Association with toxicity of polymorphisms in the 5-FU target *TYMS* and in the methylenetetrahydrofolate reductase (*MTHFR*) has been widely studied and these variants are frequently tested in pharmacogenetic assays, but there is not a general consensus about their clinical impact [15–18].

Along with pharmacogenetics, biochemical assays for pretreatment evaluation of 5-FU metabolism have been developed, including DPD deficiency testing in cell lysates from peripheral blood and the plasma measurement of uracil (U) and dihydrouracil/uracil ratio (UH_2_/U) [19–22]. The last two methods are based on the estimation of DPD activity by the level of its endogenous substrate U and the resulting metabolite, UH_2_, in plasma. This test is characterized by good sensibility of 82,4% but low specificity of 78.4%.

We previously described a pretreatment *ex-vivo* assay to determine the rate of peripheral blood mononuclear cells (PBMC) metabolizing 5-FU [23]. This parameter (individual 5-FU degradation rate, 5-FUDR) differs by others pretreatment assays, as it is not limited to the evaluation of DPD activity, but determines the net result of all the enzymatic transformation of 5-FU (Figure 1), in terms of the amount (nmol) of drug consumed by cells in a time unit. We also showed that the 5-FUDR value is consistently lower in patients who develop grade 3–4 toxicity [23].

The present study was aimed to evaluate the performance of 5-FUDR as a pretreatment predictor of grade 3–4 toxicity and to compare it with currently used pharmacogenetic markers. The distribution of allelic variants of the genes *DPYD*, *TYMS* and *MTHFR* and the pretreatment 5-FUDR was analyzed in 1010 mixed cancer patients, and the association with 5-FU toxicity was analyzed on 433 CRC patients.

## RESULTS

**Figure 2:**
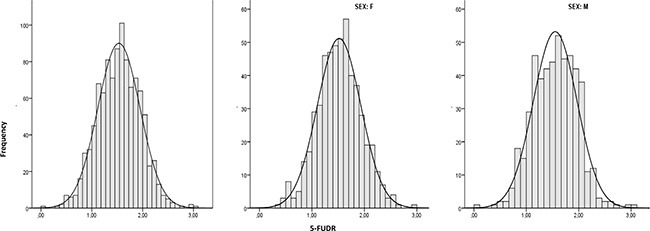
5-FU degradation rate distribution (*n* = 1010; *n* = 518, females; *n* = 492, males)

All analyzed polymorphisms were in Hardy-Weinberg equilibrium [24]. In the overall population of 1010 mixed cancer patients (51.29% females, 48.71% males, median age 66 years, age range 27–87), the mean pretreatment 5-FUDR value (± standard deviation, SD) was 1.54 ± 0.41 ng/ml/10^6^ cells/min, the median 1.55 ng/ml/10^6^ cells/min and the interquartile range 1.25–1.84 ng/ml/10^6^ cells/min (range 0.03–3.01 ng/ml/10^6^ cells/min). The departure from a normal distribution was not statistically significant (*p* = 0.82) at all, and this result was consistent with the visual inspection of the histogram and the Kernel density curve (Figure 2). The 5-FUDR parameter is not significantly affected by age, gender, cancer type, or polymorphisms in the *MTHFR* and *TYMS* genes (Table 1). Only a small difference between mean values, at the edge of significance (*p* = 0.072), appeared for the *MTHFR* A1298C genotype: the homozygous carriers of the mutant C allele have a slight decrease in mean 5-FUDR compared to AA and AC genotypes (*p* = 0.072). In contrast, and as expected, the presence of the *DPYD* IVS14+1G>A splice variant affects significantly (*p* < 0.001) the 5-FUDR, with the heterozygous carriers showing a marked decrease in the mean 5-FUDR value compared to non-carriers (0.81 ± 0.29 ng/ml/10^6^ cells/min *vs* 1.54 ± 0.41 ng/ml/10^6^ cells/min) (Table 1). The *DPYD* IVS14+1G>A polymorphisms was detected only as heterozygous with a frequency of 1.28%.

**Table 1:** 5-FU degradation rate (5-FUDR) means and 95% confidence intervals by demographic, cancer type and genetic characteristics (*N* = 1010)

		*N*	%	mean	95% CI	*p*-value*
Gender	males	492	48.71	1.55	1.51–1.59	
	females	518	51.29	1.52	1.48–1.56	0.190
Age category**	< = median	513	50.79	1.51	1.47–1.55	
	> median	495	49.01	1.56	1.52–1.60	0.100
DPYD	GG	998	98.81	1.54	1.51–1.57	
	GA	12	1.19	0.81	0.63–0.99	**< 0.001**
MTHFR1298	AA	484	48.45	1.55	1.51–1.59	
	AC	426	42.64	1.54	1.50–1.58	
	CC	89	8.91	1.44	1.35–1.53	0.072
MTHFR677	CC	285	28.70	1.52	1.47–1.57	
	CT	491	49.95	1.55	1.51–1.59	
	TT	217	21.85	1.54	1.49–1.59	0.708
Tser	2R2R	199	19.70	1.55	1.50–1.60	
	2R3R	465	46.04	1.54	1.50–1.58	
	3R3R	327	32.38	1.52	1.48–1.56	0.587
Cancer type	colon	549	54.36	1.51	1.48–1.54	
	breast	105	10.40	1.43	1.34–1.51	
	gastric	106	10.50	1.58	1.49–1.67	
	pancreas	62	6.14	1.62	1.53–1.71	
	others	188	18.61	1.52	1.42–1.63	0.102

* analysis of variance.

** for males 68/69 yrs; for females 64/65 yrs.

Due to variability in chemotherapeutic regimens among different cancer types and to statistical considerations, we proceeded to study the association of 5-FUDR and gene polymorphisms with toxicity in the most numerous cancer group, that was a subset of 433 CRC patients treated with fluorouracil based regimens. Counts and frequencies of 5-FU toxicities by demographics, specific chemotherapy regimens, genetics and pre-treatment 5-FUDR (categorized by centiles) in this subgroup are reported in Table 2. We detect a total of 92 (21.2%) cases of severe toxicity (National Cancer Institute Common Toxicity Criteria, version 3 [25], CTC3, grade 3 and grade 4, G3-G4). The *DPYD* IVS 14+1G>A splice variant was present as heterozygous with a frequency of 1.39%, comparable to the frequency in the overall population. Of the six heterozygous carriers patients in the CRC cohort, three (50.0%) developed severe toxicity, whereas 89 (20.8%) out of the 427 non-carriers developed severe toxicity. The difference in number of severe toxicity events between mutated *DPYD* and wild-type *DPYD* did not reach statistical significance (*p* = 0.113). In contrast, severe toxicity was found to be significantly associated with an age above the median (*p* = 0.007) and with a 5-FUDR below the 5th centile (called poor metabolism - PM) or above the 95th centile (called ultra-rapid metabolism - UM) (*p* = 0.002). In particular, the PM and UM are associated with a 3.47 and 3.34 increased OR, respectively, compared to the normal metabolizers (NM; 5-FUDR: 0.85–2.2 ng/ml/10^6^ cells/min).

**Table 2:** Association of severe toxicity (CTC3 Grade 3-4) with demographic, genotype, 5-FU degradation rate (5-FUDR) and chemotherapy characteristics, in the sample of colorectal cancer patients (*N* = 433) by univariate and multivariate analyses

		Total*		Grade 0–2		Grade 3–4				
		*N*	%	*N*	%	*N*	%	*p*-value**	OR^a^ (95% CI)	OR^b^ (95% CI)
Sex	males	238	54.97	192	56.30	46	50.00		1	1
	females	195	45.03	149	43.70	46	50.00	0.281	1.29 (0.81–2.04)	1.47 (0.88–2.45)
Age category***	< = median	205	47.34	173	50.73	32	34.78		1	1
	> median	228	52.66	168	49.27	60	65.22	**0.007**	2.39 (1.43–4.01)	2.41 (1.41–4.12)
DPYD	GG	427	98.61	338	99.12	89	96.74		1	1
	GA	6	1.39	3	0.88	3	3.26	0.113	3.80 (0.75–19.14)	4.78 (0.71–32.19)
MTHFR1298	AA	206	48.02	156	46.15	50	54.95		1	1
	AC	183	42.66	150	44.38	33	36.26		0.69 (0.42–1.12)	0.53 (0.29–0.98)
	CC	40	9.32	32	9.47	8	8.79	0.317	0.78 (0.34–1.80)	0.68 (0.23–2.04)
MTHFR677	CC	122	28.57	99	29.46	23	25.27		1	1
	CT	209	48.95	158	47.02	51	56.04		1.39 (0.80–2.42)	1.26 (0.64–2.49)
	TT	96	22.48	79	23.51	17	18.68	0.305	0.93 (0.46–1.85)	0.62 (0.25–1.56)
TYMS TSER	2R2R	83	19.53	65	19.29	18	20.45		1	1
	2R3R	198	46.59	154	45.70	44	50.00		1.03 (0.55–1.92)	1.01 (0.52–1.99)
	3R3R	144	33.88	118	35.01	26	29.55	0.624	0.80 (0.41–1.56)	0.74 (0.36–1.53)
5-FUDR	NM (5–95 centile)	393	90.76	318	93.26	75	81.52		1	1
	PM (< 5centile)	19	4.39	10	2.93	9	9.78		3.82 (1.50–9.72)	3.47 (1.19–10.10)
	UM (> 95centile)	21	4.85	13	3.81	8	8.70	**0.002**	2.61 (1.04–6.52)	3.34 (1.27– 8.83)
Chemotherapy	mFolfox6	177	40.97	133	39.12	44	47.83		1	1
	Xelox	46	10.65	33	9.74	13	14.13		1.19 (0.58–2.46	1.26(0.57–2.82)
	Capecitibine	131	30.32	111	32.65	20	21.74		0.54 (0.30–0.98)	0.35 (0.18–0.69)
	mFolfiri	64	14.81	50	14.71	14	15.22		0.85 (0.43–1.68)	0.83 (0.40–1.71)
	5-fluorouracil	14	3.24	13	3.82	1	1.09	0.126	0.23 (0.03–1.83)	0.17 (0.02–1.77)

Abbreviations: OR, odds ratio; CI, confidence interval; NM, normal metabolism; PM, poor metabolism; UM, ultra metabolism.

* totals may vary because of missing value.

** Chi squared test or Fisher exact test

*** cutoff median value for males: 68/69; for females: 64/65

a crude OR

b adjusted OR.

Using the 5-FUDR value as a stratification factor to identify patients at higher risk of 5-FU G3-G4 toxicity (e.g. PM or UM subjects), 40 patients (9.24% of total population) potentially at risk can be recognized, of whom 17 (42.5%) really developed G3-4 toxicity. Using the presence of the *DPYD* IVS 14+1G>A splice variant as a risk predictive factor, we could detect only 6 patients (1.39% of total population) carriers of the marker, of whom 3 (50.0%) really developed G3-G4 toxicity. Sensitivity, specificity, positive and negative predictive values (PPV and NPV) of the *DPYD* IVS 14+1G>A polymorphisms and of the 5-FUDR metabolic classes (PM, UM, and PM plus UM) are reported in Table 3.

**Table 3:** Positive predictive value (PPV), negative predictive value (NPV), sensitivity and specificity of the 5-FU degradation rate (5-FUDR) test and the DPYD IVS14+1G> A genotyping test

	PPV	NPV	Sensitivity	Specificity
**5-FUDR PM+UM**	42.50	80.92	18.48	93.26
**5-FUDR PM**	47.37	80.92	10.71	96.95
**5-FUDR UM**	38.10	80.92	9.64	96.07
**DPYD**	50	79.16	3.26	99.12

Abbreviations: PM, poor metabolism; UM, ultra-rapid metabolism.

## DISCUSSION

In recent years, pharmacogenetics has been regarded as the most promising tool for preemptive risk stratification, but recent results from large studies spread some doubts about the actual usefulness of this approach. [26–28].

This is the reason why alternative roads should be walked to overcome the limits of 5-FU pharmacogenetics. 5-FU represents an elective drug to attempt biochemical approaches in predicting drug metabolism. In fact, unlike many drugs, the main metabolizing enzymes are expressed in peripheral blood cells, allowing to evaluate the efficiency of the individual drug metabolism by non-invasive procedures such as using a peripheral blood sample. Pre-treatment determination of DPD enzymatic activity in cell lysates from peripheral lymphocytes and the plasma assessment of UH_2_/U ratio are well established assays [7]. Previously, we developed a related biochemical assay aimed to evaluate the 5FU-metabolism preemptively, but with a significant functional difference. Whereas the above mentioned tests are targeted to determine the activity level of the DPD enzyme, the 5-FUDR assesses the combinatorial effects produced by all the 5-FU metabolizing enzymes, both activating (i.e.: orotate phosphoribosyltransferase, OPRT; thymidylate phosphorylase, TP) and inactivating (i.e.: dihydropyrimidine dehydrogenase, DPD) (Figure 1). Hence, the 5-FUDR parameter translates the effects of known and unknown genetic determinants, affecting 5-FU metabolizing enzymes, into a measurable phenotype. In this study, we have evaluated the general characteristics of the pretreatment 5-FUDR value on more than 1000 subjects, showing that it is a continuous parameter with a normal distribution in the population. This observation is consistent with the known existence of a fraction of persons with a very low rate of 5-FU metabolism (PM), but also highlights the existence of a class of subjects with an extremely rapid 5-FU metabolism (UM).

The effect of the individual 5-FUDR on 5-FU toxicity was analyzed in a subgroup of 433 CRC patients. The total number of severe toxicities observed in this group (*n* = 92, 21.2%), as well as the higher frequency in older patients, are consistent with the data reported in the literature [12, 13].

Strikingly, we have found that both the PM subjects, defined by a 5-FUDR <= 5th centile, and the UM subjects, defined by a 5-FUDR > 95th centile, are at higher risk of developing G3-4 toxicity, with an OR of 3.47 and 3.34, respectively, compared to the class of normal metabolizers (5th < 5-FUDR < =95th centiles). While a higher percentage of toxicity in PM was expected, the association of 5-FU UM with severe toxicity was more surprising. Indeed, to our knowledge, no similar relationship between toxicity and a fast drug metabolism has been reported, probably due to the lack of analytical tests able to identify this kind of ultra-metabolizers. The potential clinical consequences of a 5-FU ultra-rapid metabolism are intriguing and deserve further studies. In fact, the mechanism underlying a faster rate of 5-FU consumption (that is, a higher 5-FUDR value) may involve an increased activity of DPD or an increased activity of the enzymes producing the active drug metabolites (Figure 1). This hypothesis is consistent with data showing that the 5-FU sensitivity is correlated with polymorphisms in the OPRT gene as well as in cancer tissues with the level of activity of the OPRT enzyme and with the OPRT/DPD activity ratio [29–32]. Thus, it could be speculated that the individual 5-FUDR may be related to progression free survival (PFS) and overall survival (OS), due to an increased fraction of cytotoxic metabolites in ultra-metabolizers.

As reported in Table 3, in the analyzed CRC patients cohort, the 5-FUDR test allows risk stratification with a specificity and a NPV similar to that obtained using the *DPYD* IVS 14+1G>A genotyping and with a PPV ranging from slightly to moderately lower than the *DPYD* IVS 14+1G>A PPV (47.37% for 5-FUDR PM and 38.10% for 5-FUDR UM compared to 50% of genotyping). In contrast, the sensitivity of the 5-FUDR test is much higher than the DPYD genotyping (18.48% *vs* 3.26%).). Even though it still is far from the ideal preemptive assay, this increase in sensitivity means that we would have had the opportunity to avoid14 more cases of severe (potentially lethal) toxicity compared to the 3 cases correctly predicted by the *DPYD* genotyping (Table 2). The advantage of the 5-FUDR test consists in the higher prevalence of the markers used to identify patients with poor and ultrarapid metabolism, which by definition consist of a 10% of the population (5-FUDR < 5th centile and > 95th centile) compared to the 1.28% frequency of the *DPYD* IVS 14+1G>A polymorphisms (frequency in the 1010 Italian patients sample).

However, proposal for novel preemptive tests must consider cost-effectiveness. In the case of 5-FUDR, the cost of the assay per sample is quite low (estimated at around $10), since it does not required commercial kit but it is based on the chromatographic separation of the analyte, which is also a method easily transferrable into clinical laboratories. Considering that the cost derived by inpatient care of each G3-G4 toxicity is generally calculated in hundreds of dollars [33], the 5-FUDR assessment is supposed to be cost-effective. Further, the test result is available within one working day, so the test can easily be scheduled during the pre-treatment phase of patient evaluation and therapy selection.

A limitation for this study is that the pharmacogenetics assay for prediction of 5-FU toxicity includes only the main *DPYD* splice site polymorphisms, while additional functional polymorphisms have recently been proposed as important markers.

The future direction in the field of preemptive identification of severe toxicity during 5-FU treatment should point to the combination of genetic and phenotypic markers to improve the sensitivity for detection of patients at risk. The individual 5-FUDR appears as a suitable marker for this scope. Besides, the 5-FUDR test gives the opportunity to evaluate the potential clinical implications of the 5-FU ultra rapid metabolism and, in our opinion, it deserve further investigation as a prognostic markers of 5-FU treatment.

Finally, the significant number of severe toxicity currently undetectable by preemptive assays, highlights the need for a deeper understanding of the fluorouracil metabolism. Estimating the ratio between active and inactive drug metabolites probably will be the key to uncover multiple mechanisms mediating 5-FU efficacy and toxicities.

## MATERIALS AND METHODS

### Patients

Data collected from 1010 cancer patients followed at the Sant'Andrea Hospital of Rome, Italy, between April 2009 and April 2013, was analyzed in this retrospective study. Inclusion criteria were: age > 18 years; histologically documented cancer; Eastern Cooperative Oncology Group performance status 2 or less; cancer therapy toxicity absent; written informed consent. Exclusion criteria: relevant diseases within 6 months (i.e.: myocardial infarction, lung fibrosis, etc); 5-FU based chemotherapy in the past. Chemotherapy-related toxicity in the first six cycles of treatment was recorded according to National Cancer Institute Common Toxicity Criteria, version 3 (CTC3) [25].

To study associations between DNA polymorphisms, 5-FUDR and 5-FU-related toxicity, we have selected a subset of 433 CRC patients out of 1010 whose therapy was based essentially on fluorouracil, e.i.: mFOLFOX6, mFOLFIRI, XELOX with or without bevacizumab or cetuximab, and capecitabine.

The study was conducted in accordance with the Declaration of Helsinki and the protocol was approved by the institutional (Sapienza University) ethical committee (Rif. 3762_2015/23.07.2015, Prot. 2377/2015).

### Genotyping

Germinal polymorphisms were analyzed as follows: genomic DNA was isolated from peripheral blood using the X-tractor Gene system (Corbett Life Science, Australia). The splice-site polymorphism, IVS14+1G>A in the *DPYD* gene, C677T and A1298C SNPs in *MTHFR* gene were analyzed using the commercial kit for fluoropyrimidine response (Diatech, Jesi, Italy) according to manufacturer's protocol; briefly, region covering the SNP of interest was amplified by PCR using specific primers, and then sequenced using the Pyrosequencer PyroMark ID system (Biotage AB and Biosystems, Uppsala, Sweden). The variable number of tandem repeats (VNTR; 2R or 3R) in the *TYMS* enhancer region (TSER) was determined by the Polymerase Chain Reaction (PCR) according to manufacturer's protocol (fluoropyrimidine response - Diatech, Jesi, Italy) and visualized onto 2.2% agarose gel.

### Determination of the individual 5-FU degradation rate

The test was performed as previously reported [23], using a HPLC-MS/MS instrument (*high performance liquid chromatography-mass spectrometry/mass spectrometry)* including an Agilent 1100 chromatographic system coupled to an API 3200 triple quadrupole (ABSCIEX, Framingham, MA, USA). Briefly, freshly prepared peripheral blood mononuclear cells (2.5–3.5 × 10^6^ cells) are incubate at 37°C, with shaking, with a known amount of 5-FU. Cells aliquots are drawn at time 0, 1 h and 2 h, lysed and centrifuged and the concentration of 5-FU in the supernatants is quantified by HPLC-MS/MS. The 5-FUDR is expressed as ng 5-FU/ml/10^6^ cells/min.

### Statistics

Statistical analyses were performed using STATA, version 11.0 (StataCorp, College Station, Tex).

Shapiro-Wilk test was used as formal test for departure from a normal distribution, and histogram with a normal curve and a kernel density curve overlaid was also performed for visual inspection of 5-FUDR and age data distribution.

To remove some variability in outcome -at all covariate values-, while maintaining structure of the relationship between outcome and independent variables, the independent variables were categorized as follow. Subjects were subdivided into two groups with respect to median age. The 5-FUDR parameter was divided into 6 groups according with the value of the 5th, 25th, 50th, 75th, and 95th centile (≤ 0.85; > 0.85 ≤ 1.25; > 1.25 ≤ 1.55; > 1.55 ≤ 1.84; > 1.84≤2.2; > 2.2 ng/ml/10^6^ cells/min, respectively). For further analysis on the CRC patients subset, the data between 0.86 and 2.2 ng/ml/10^6^ cells/min (5th–95th centiles) were taken together to create a reference group (NM, normal metabolisms). Dummy code was applied to sex, female = 1 and male = 0 in regression analysis.

Data about symptoms’ severity were dichotomized as no/mild toxicities (CTC3 grade 0, 1 and 2) versus severe toxicity (CTC3 G3-4).

Data, presented as proportions and differences between groups, were tested with Chi-squared or Fisher exact test. Univariate and multivariate odds ratios (ORs), and associated 95% confidence intervals (CIs) for potential variables associated with severe toxicities were estimated using logistic regression models. We used the following model-building process: first we assessed bivariate associations between the dependent variable and each of the potential covariates; covariates not significantly associated (*p* > 0.10) with the outcome were dropped from further consideration in modeling outcome. The remaining candidate covariates were entered into a multiple regression model and subjected to backward selection until all remaining covariates had *p*-value < 0.05, adjusted for the other remaining covariates. Gender, age, and gene polymorphisms were treated as confounding variables. Akaike's information criteria and the likelihood ratio test were used to define the multivariable model.

Deviations from Hardy-Weinberg equilibrium were assessed using the online HWE test calculator at http://www.oege.org/software/hwe-mr-calc.shtml [24].

## Abbreviations

5-FU: 5-fluorouracil
5-FUDR: 5-FU degradation rate (expressed as ng 5-FU/ml/10^6^ cells/min)
CI: confidence interval
CRC: colorectal cancer
CTC3: National Cancer Institute Common Toxicity Criteria, version 3
DHFU: 5, 6-dihydrofluorouracil
DHP: dihydropyrimidinase
DPD: dihydropyrimidine dehydrogenase
DUT: dTUPase
FBAL: α-fluoro-β-alanine
FdUDP: fluorodeoxyuridine diphosphate
FdUMP: fluorodeoxyuridine monophosphate
FdUTP: fluorodeoxyuridine triphosphate
FUDP: fluorouridine diphosphate
FUDR: 5-fluoro-2′-deoxyuridine;
FUMP: fluoroyuridine monophosphate
FUPA: α-fluoro-β-ureidopropionic acid
FUR: 5-fluorouridine
FUTP: fluorouridine triphosphate
G3: grade 3 toxicity
G4: grade 4 toxicity
HPLC: high performance liquid chromatography
MS: mass spectrometry
MTHFR: methylenetetrahydrofolate reductase
NM: normal metabolism
NPV: negative predictive value
OPRT: orotate phosphoribosyltransferase
OR: odds ratio
OS: overall survival
PBMC: peripheral blood mononuclear cells
PCR: Polymerase Chain Reaction
PFS: progression free survival
PM: poor metabolism
PPV: positive predictive value
QUASAR2: Quick and Simple and Reliable study
RRM2: ribonucleotide reductase
SD: standard deviation
TK: thymidine kinase
TP: thymidylate phosphorylase
TSER: thymidylate synthase enhancer region
TYMS: thymidylate synthase
U: uracil
UCK1: uridine diphosphate kinase
UH2: dihydrouracil
UK: uridine kinase
UM: ultra-rapid metabolism
UMPK: uridine monophosphate kinase
UPB: β-ureidopropionase
UPP1: uridine phosphorylase
VNTR: variable number of tandem repeat
